# Shift work schedule patterns and sleep outcomes: results from the SHIFT2HEALTH survey across eight European countries

**DOI:** 10.5271/sjweh.4329

**Published:** 2026-11-01

**Authors:** Isabel Santonja, Coen Dros, Sandra Haider, Eva Winzer, Maria Wakolbinger, Katrin Scionti, Vanessa Schoissengeier, Magdalena A Wrzesińska, Katarzyna W Binder-Olibrowska, Jarosław Rakoczy, Anna Lena Aufschnaiter, Christina Höfler, Marlies Wallner, Catalina Cuparencu, Karl-Heinz Wagner, Hendriek C Boshuizen, Monique H Vingerhoeds, Heidi Lammers-van der Holst, Kyriaki Papantoniou

**Affiliations:** 1 Medical University of Vienna, Centre for Public Health, Department of Epidemiology, Vienna, Austria.; 2 Erasmus University Medical Center, Department of Public health, Rotterdam, The Netherlands.; 3 Medical University of Vienna, Centre for Public Health, Department of Social and Preventive Medicine, Vienna, Austria.; 4 University of Vienna, Department of Nutritional Sciences, Vienna, Austria.; 5 Vienna Doctoral School of Pharmaceutical, Nutritional and Sport Sciences (PhaNuSpo), Vienna, Austria.; 6 Medical University of Lodz, Department of Psychosocial Rehabilitation, Lodz, Poland.; 7 FH JOANNEUM, University of Applied Sciences, Institute of Dietetics and Nutrition, Graz, Austria.; 8 University of Copenhagen, Department of Nutrition, Exercise and Sports, Copenhagen, Denmark.; 9 Biometris, Wageningen University and Research, Wageningen, The Netherlands.; 10 Wageningen Food & Biobased Research, Wageningen, The Netherlands.; 11 Barcelona Institute for Global Health (ISGlobal), Barcelona, Spain.

**Keywords:** insomnia, night shift work, rotating shift work, sleep duration, sleep quality

## Abstract

**Objectives:**

This study aimed to describe shift work patterns in Europe using a data-driven approach and evaluate their impact on adverse sleep outcomes.

**Methods:**

We analyzed 6245 participants from the SHIFT2HEALTH survey across eight European countries, who provided information on work schedules, shift work metrics, sleep quality and timing (MCTQshift) across different shifts, and chronic insomnia (Insomnia Severity Index). We used mixtures of von Mises-Fisher models to identify shift clusters based on shift start and end times and evaluated differences in shift-specific sleep duration and quality using Bayesian multilevel models. Associations of shift metrics with short average sleep duration and chronic insomnia, adjusting for confounders, were evaluated using Bayesian logistic regression.

**Results:**

We identified five shift clusters: day, evening and night (mean shift lengths: 7.8, 7.9 and 9.5 hours, respectively), long day and long evening (mean lengths: 10.5 and 12.8 hours, respectively). Sleep duration and quality varied between clusters, with the shortest duration [mean 6.00, 95% credible interval (CI) 5.95–6.06 hours] and poorest quality (29.3% “poor”/“very poor”) sleep reported between night shifts. Compared to day workers, greater chronic insomnia odd ratios (OR) were observed among participants with >90% night shifts (OR 1.26, 95% CI 1.01–1.58), rotating shifts (OR 1.42, 95% CI 1.22–1.65) and longer night work history (OR_≥16 years_ 1.28, 95% CI 1.08–1.52). These variables and longer weekly working hours [OR _6.10h increase_ 1.13, 95% CI 1.04–1.22) were associated with short sleep.

**Conclusions:**

Data-driven analyses of work schedules may provide a more comprehensive evaluation of the impact of real-world shift work patterns on sleep.

Approximately 20% of the European Union (EU) workforce engages in some form of shift work (1). Working irregular hours, including nights, can lead to circadian misalignment between endogenous rhythms and externally imposed sleep-wake schedules (2)

Around one in four shift workers could develop Shift Work Sleep Disorder (3), a condition characterized by clinically relevant insomnia symptoms and/or sleepiness, and impaired daytime functioning (4). In addition, current night shift workers commonly experience a variety of sleep disturbances, such as reduced sleep duration and quality (5, 6). Previous research showed that night shift work is associated with chronic insomnia and daytime sleepiness, even after the cessation of night shift work, particularly among those reporting a high frequency of night shifts (7). Furthermore, current night shift workers commonly experience a variety of sleep disturbances, such as reduced sleep duration, poor sleep quality, difficulties initiating or maintaining sleep, or waking up too early.

Optimizing shift schedules may reduce the risk of sleep problems, which is critical for workers’ safety and health. Long working hours and night shifts have been associated with a higher incidence of accidents, with sleep proposed as a mediator of this association (8, 9). A recent Cochrane review (10) of 11 studies showed that interventions targeting shift rotation speed or direction or shift length could improve sleep. However, the evidence from these interventions was rated low to very low, partly due to methodological limitations, highlighting the need of high-quality studies.

A key limitation of most existing interventions (10) and observational studies [eg (7–9, 11–13),] is that they have examined separately individual shift characteristics that co-occur in real-world settings (eg, analyzing shift start or end time separately from shift length) and, thus, may fail to capture the complexity of real-world schedules. In contrast, data-driven approaches, such as cluster (14, 15) or latent class analysis (16), offer a promising alternative by simultaneously accounting for multiple schedule features and identifying naturally occurring shift patterns. This underscores the need for multi-feature approaches to comprehensively characterize the impact of shift schedules on workers’ sleep health and to guide future interventions and scheduling recommendations.

Thus, our aims were threefold: (i) describe shift work schedule patterns in a large European sample, (ii) examine the impact of shift clusters on shift-specific sleep duration and sleep quality (shift-level), and (iii) explore the association between classic shift work metrics (such as night shift frequency and years worked on night shifts) and the occurrence of chronic insomnia and short sleep duration (participant-level).

## Methods

### Study design and population

As part of the Horizon Europe-funded SHIFT2HEALTH project (www.shift2health.eu), an online survey was conducted from May 2024 to January 2025 in Austria, Denmark, Germany, Greece, Italy, The Netherlands, Poland and Spain. It was available in local languages and English and promoted via social media, professional networks, and a social research agency. It took 15–20 minute to complete and elicited information on sociodemographics, anthropometry, work schedules, sleep, diet, physical activity, and well-being.

Participants were eligible if they were ≥21 years, (self-)employed, and worked ≥28 hours/week. Of 13 469 eligible respondents, 6245 participants were included in this analysis, after excluding those with missing information on key variables or reporting extreme shift lengths (<3 or >25 hours) or average sleep duration (<3 hours) (supplementary material, www.sjweh.fi/article/4329 figure S1).

Survey data were collected and managed using REDCap (Research Electronic Data Capture) hosted at the University of Vienna (17, 18).

### Ethical approval

This study was conducted in accordance with the Declaration of Helsinki. Participation was voluntary and anonymous. The Research Ethical Committee of Wageningen University & Research, The Netherlands, obtained ethical approval for all countries (2024-001, 25-01-2024). Specific approvals were obtained for Austria (Ethics commission, Medical University of Vienna; EK Nr: 1152/2024) and Germany (Ethics commission, University of Bremen; Aktenzeichen: 2024-04).

### Variables

*Work schedules.* Participants reported shift start and end times (24-hour format) and monthly frequency for up to four different current work shifts. Shift length (hours) was calculated from start and end times and checked by participants. Prior to analysis, shifts were classified as: early (starting before 06:00 hours and not meeting the definition of night shift), day (starting 06:00–09:59 hours), evening (starting at 10:00 or later and ending before 02:59 hours) or night shifts (≥3 hours of work during 00:00–05:00 hours). Participants’ schedules were classified as “permanent day”, “permanent night” and “rotating shifts”.

Monthly shift frequency was transformed to relative frequency (%), to account for errors in data entry (eg, participants reporting weekly instead of monthly frequency). The relative frequencies of the different shift clusters were computed for each participant and categorized into >0–15, >15–30, >30–50, >50–70, >70–90 and >90%.

*Classic night shift work metrics*. Relative frequency of night shifts (prespecified category) was calculated in the same way as the relative frequencies of the shift clusters. The survey also elicited information on night shift work status (always day worker, former or current night shift worker), speed of shift rotation (daily change, change every 2–4 days, change every week, change every 2–3 weeks, other, or rotation speed varies), years worked on night shifts (<1, 1–5, 6–15, and ≥16 years) and weekly working hours (ie, total hours worked in a week). A schedule type variable with three categories (permanent day, permanent night, and rotating shifts) combined information of current schedule (current night workers versus other) and number of shift types (1 versus ≥2).

*Sleep.* To assess sleep timing, we modified the ultra-short Munich Chronotype Questionnaire (micro-MCTQ) (19) by asking sleep timing questions tailored to reported shift hours, similar to the MCTQ for shift workers (MCTQShift) (20). For each reported shift, participants provided their typical sleep and wake times between two consecutive shifts. Participants who did not work two consecutive shifts on the same schedule, were asked to report their sleep habits after this type of shift or before in the case of early shifts (shifts starting before 10:00 hours).

Shift specific sleep duration (hours) was calculated as the time elapsed between sleep- and wake-up time for work days with different shift schedules and days-off separately. Shift-specific sleep quality was derived from a question in which participants were asked to rate their sleep quality between two identical shifts for each shift reported using a Likert scale (“very poor”, “poor”, “moderate”, “good” or “very good”).

Average sleep duration on workdays was calculated as the frequency-weighted average of sleep durations when working a specific type of shift. Average sleep duration was then calculated as:* *

From this, a binary variable (*short average sleep duration*) was calculated, using a cut off of <6 hours (21).

We used the first and third questions of the Insomnia Severity Index (22) to elicit information on insomnia symptoms (ie, difficulties falling or staying asleep or problems waking up too early). Participants reporting insomnia symptoms were asked about the frequency and duration of symptoms and associated daytime impairment. Chronic insomnia was considered present if participants reported mild-to-very severe insomnia symptoms occurring ≥3 times/week for ≥3 months, and associated daytime impairment (“somewhat”, “much” or “very much interfering”).

*Covariates.* The survey assessed sociodemographic characteristics (age, gender, country of residence, marital status, education level and income satisfaction), work sector, height, weight, chronotype (23) and tobacco and alcohol consumption. Body mass index (BMI) was calculated from self-reported height and weight. Missing values for country of residence (N=95), smoking status (N=195) and alcohol consumption (N=195) were labeled “unknown”. Missing values for BMI (N=15) were imputed using the median (25.0 kg/m^2^).

### Statistical analysis

*Description of shift schedules.* To group similar shifts, we used a data-driven clustering approach. Concretely, we applied mixtures of von Mises-Fisher (VMF) model-based clustering using the moVMF R-package (24). VMF clustering is an algorithm that groups data points on a multi-dimensional sphere by using the VMF distribution. This distribution is particularly well suited for representing the 24-hour day, as it accurately measures the true directional proximity of different times of the day, such as shift start and end time.

Each shift was represented by its start and end times. These times were transformed into radians, decomposed into sine and cosine components and concatenated into a single vector of four components [ie, sin(start time), cos(start time), sin(end time), cos(end time)]. This vector was then normalized to unit length to satisfy the requirements of the VMF distribution. Because this four-component vector mathematically encapsulates both the timing and the length of each shift, no additional features or covariates were included in the clustering model.

We tested solutions for *k*=1 to ten clusters and selected *k*=5, as it presented adequate levels across all of the diagnostic metrics considered [Bayesian Information Criterion (BIC), silhouette score, k-fold cross-validation and Adjusted Rand Index (ARI)] (supplementary figure S2) and resulted in clearly interpretable and distinct shift clusters. Cluster groups were named according to shift start time and length (eg, long day shift).

*Comparison of shift-specific sleep characteristics*. To compare sleep duration and quality across shift clusters while accounting for multiple measures per participant, Bayesian multilevel linear (sleep duration) and ordinal (sleep quality) models were used (supplementary appendix 1 and table S1). We chose a Bayesian framework for two primary reasons. First, it yields 95% credible intervals (CI), which provide the actual probability that the true parameter lies within the specified range given the data. This offers a more intuitive epidemiological interpretation than frequentist confidence intervals, which describe the hypothetical long-run reliability of the method rather than the probability of the actual parameter. Second, we evaluated differences across shift clusters—using day shifts as the reference—based on the probability of direction (p-direction). The p-direction quantifies the certainty that an effect is consistently positive or negative; for instance, a p-direction of 0.99 indicates a 99% probability that sleep duration or quality is truly higher (or lower) in that shift cluster compared to day shifts. We considered effects with a p-direction ≥0.975 to provide credible evidence of a difference. To examine whether associations between shift clusters and sleep outcomes varied by chronotype, we added interaction terms between chronotype and shift clusters in the models.

A*ssociation of shift cluster schedule composition and shift metrics with sleep outcomes.* The association between shift cluster schedule composition or night shift metrics with sleep outcomes (chronic insomnia and short average sleep duration) was modeled directly using Bayesian logistic models. The Bayesian approach allowed us to incorporate known outcome prevalences using informative intercept priors, while applying weakly informative priors to covariates (see supplementary appendix 1 and table S1). Prior specification, a key feature of the Bayesian framework, regularizes estimates by shrinking coefficients toward a conservative baseline, thereby reducing the risk of overfitting and ensuring stable, reliable risk estimates [odds ratios (OR)]. Furthermore, similarly to our sleep models, the resulting 95% CI represent the actual 95% probability that the true risk parameter falls within the estimated bounds given the observed data, rather than reflecting long-run frequencies.

First, to evaluate the impact of reallocating working time from day shifts to other shift types, we performed an isotemporal substitution analysis. These models used categorized shift frequencies as the primary exposure, with 100% day shifts as the reference. This structure ensured total work time remained constant (100%), so any increase in a specific shift cluster represented the displacement of an equivalent proportion of day shifts. We defined three levels of adjustment: model 1 (M1= adjusted for age and sex), model 2 (M2=M1 additionally adjusted for chronotype, country of residence, education, income satisfaction, marital status, BMI, alcohol consumption and smoking status) and model 3 (M3= M2 further adjusted for years worked on night shifts and weekly working hours).

Lastly, we analyzed classic night shift metrics (night shift status, years worked on night shifts, type of shift schedule, frequency of night shifts, rotation speed and weekly working hours) as independent exposures. For each exposure-outcome combination, we fitted M1 and M2. An additional mutually adjusted model (M4) included the variables years worked on night shifts, schedule type and weekly working hours simultaneously.

Secondary analysis included complete-case analysis, models using non-informative priors (supplementary table S1) and stratification by chronotype.

Covariates were selected a priori based on the literature and a directed acyclic graph (supplementary figure S3). Continuous covariates were scaled prior to modeling. Further information on model specifications, prior selection and model diagnostics is provided in supplementary appendix 1 and table S1.

Statistical analysis were performed with R 4.3 version.

## Results

In the final sample (N=6245), 50.7% of participants were female. Average age and BMI were 40.6 (SD 10.7) years and 26.0 (SD 5.3) kg/m^2^. Most participants lived in Austria (15.9%), The Netherlands (13.5%), Poland (12.9%) and Germany (12.6%), worked in the health, social work, beauty care sector (42.1%), had pursued university education (44.2%) and did not report difficulties living with their income (78.4%). A description of the sample is provided in supplementary table S2.

### Shift schedules clusters versus pre-defined shift categories

Participants provided information on 12 104 distinct shifts; 45.8% of participants had permanent schedules, while 21.8%, 25.2% and 7.2% reported two, three and four different shifts. According to the prespecified (ie, not data-driven) definitions, 343 (2.8%) shift schedules were classified as early, 5119 (42.3%) as day, 2573 (21.3%) as evening and 4069 (33.6%) as night shifts (table 1).

**Table 1:** Description of shift work schedules in the sample according to prespecified categories and the final Van Mises cluster solution (k=5). [SD=standard deviation.]

	N (%)	Start time		End time		Shift length (hours)		Pre-specified shift category						
								Early shifts		Day shifts		Evening shifts		Night shifts
		Mean (SD)		Mean (SD)		Mean (SD)		N (%)		N (%)		N (%)		N (%)
Prespecified categories														
Early shifts	343 (2.8)	04:52 (0:53)		13:39 (2:37)		9.1 (2.8)		343 (100.0)		0 (0.0)		0 (0.0)		0 (0.0)
Day shifts	5119 (42.3)	07:16 (0:55)		16:00 (2:07)		8.9 (2.2)		0 (0.0)		5119 (100.0)		0 (0.0)		0 (0.0)
Evening shifts	2573 (21.3)	14:06 (1:47)		21:52 (1:51)		7.7 (1.7)		0 (0.0)		0 (0.0)		2573 (100.0)		0 (0.0)
Night shifts	4069 (33.6)	21:14 (3:11)		06:52 (1:29)		11.0 (4.6)		0 (0.0)		0 (0.0)		0 (0.0)		4069 (100.0)
Cluster groups														
Day shifts	3399 (28.1)	06:58 (0:55)		14:43 (0:56)		7.8 (0.94)		197 (5.8)		3201 (94.2)		0 (0.0)		1 (0.0)
Evening shifts	1767 (14.6)	14:20 (0:41)		22:16 (0:44)		7.9 (0.64)		0 (0.0)		0 (0.0)		1766 (100.0)		0 (0.0)
Night shifts	3473 (28.7)	21:14 (1:47)		06:45 (1:01)		9.5 (2.04)		0 (0.0)		0 (0.0)		1 (0.0)		3472 (100.0)
Long day shifts	1984 (16.4)	07:43 (1:07)		18:14 (1:10)		10.5 (1.85)		30 (1.5)		1833 (92.4)		119 (6.0)		2 (0.1)
Long evening shifts	1481 (12.2)	11:36 (5:08)		01:39 (8:10)		12.8 (7.42)		116 (7.8)		85 (5.7)		686 (46.4)		594 (40.2)

The five-cluster solution identified day, evening, and night shifts groups (lengths: 7.8, 7.9 and 9.5 hours, respectively), similar to prespecified categories but with less variation in timing and duration. Two additional clusters – long day (mean length 10.5 hours) and long evening shifts (mean length 12.8 hours) – captured shifts previously categorized mostly as day, evening, or night shifts that exceeded average lengths (table 1).

### Shift-specific sleep characteristics

We found strong evidence that sleep duration varied between shift clusters (p-direction >0.99, figure 1A) compared to day shifts (mean 6.85, 95% CI 6.80–6.90). The shortest sleeps were observed between night shifts (mean 6.00, 95% CI 5.95–6.06 hours) and the longest between evening shifts (mean 7.91, 95% CI 7.84–7.98 hours). Sleep quality reports (figure 1B) between day shifts were mostly favorable (86.3% moderate-to-very-good sleep quality). Compared to day shifts, participants reported worse sleep quality between night shifts (70.7% moderate-to-very-good sleep quality) and better between both evening and long day shifts (94.5% and 88.7% moderate-to-very-good sleep quality, respectively).

**Figure 1:**
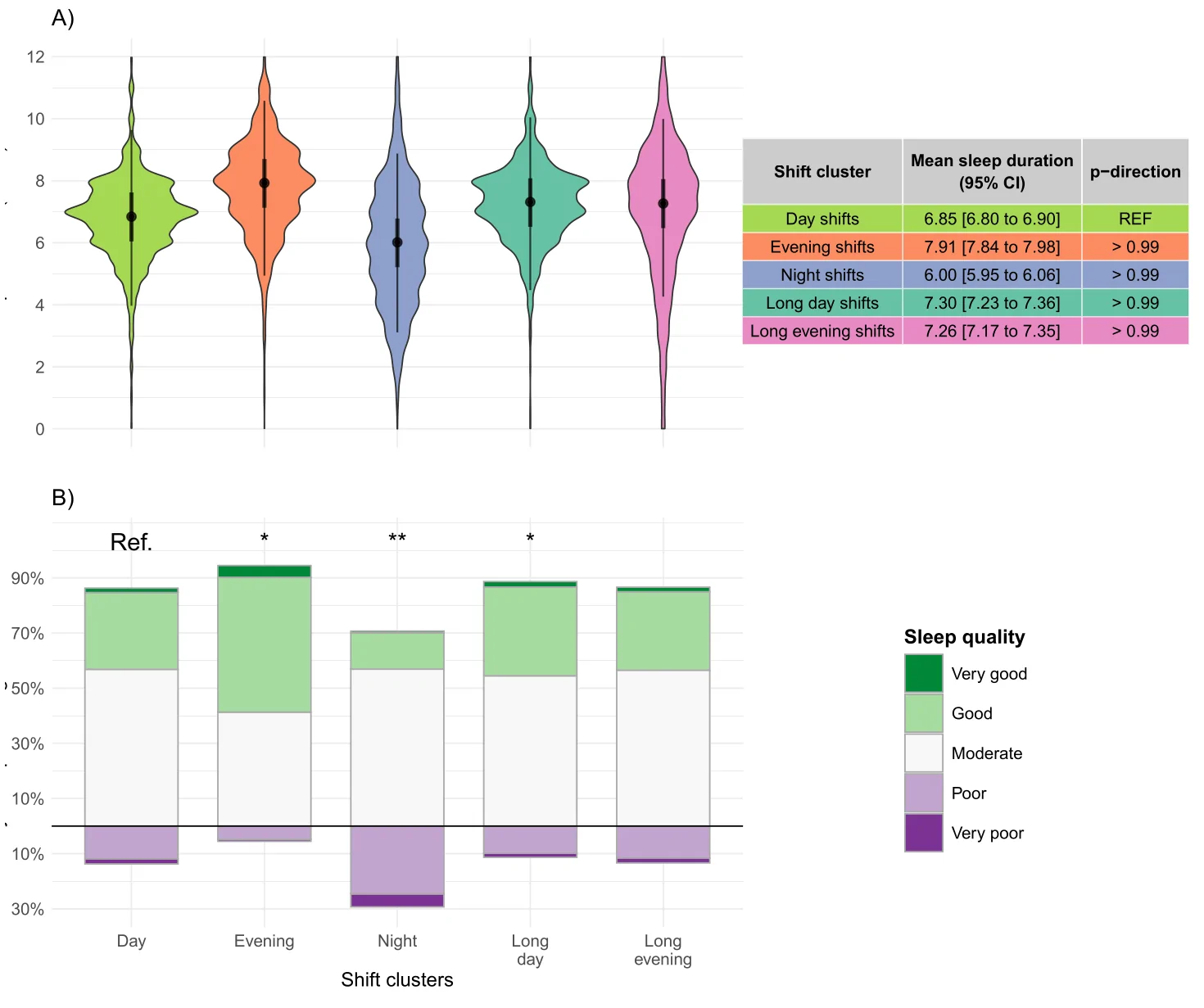
Shift-specific sleep duration (A) and quality (B) across data-driven shift clusters (N=12 104 shifts from 6245 subjects). All estimates were derived from a Bayesian multilevel models accounting for repeated measures within subjects. A) The violin plots represent the observed distribution of sleep duration for each shift cluster. Overlaid on each violin is the Bayesian posterior predictive interval: the central dot represents the posterior median, the thick bar indicates the 50% credible interval (CI), and the thin lines represent the 95% CI. The associated table showcases predicted mean sleep duration for each shift (95% CI), and the probability of direction (p-direction), which indicates the certainty that the shift is associated with longer or shorter sleep (eg, 1.00 indicates 100% certainty). B) Diverging stacked bars representing the probability of sleep quality categories derived adjusted for repeated measures using an ordinal mixed-effects model. Day shifts: reference category (Ref.). * and ** indicate a 99% probability of sleep quality being better or worse, respectively, compared to between day shifts.

Chronotype modulated shift-specific sleep duration and sleep quality (supplementary figure S4). Early chronotypes reported longer sleep and better sleep quality than intermediate and late chronotypes between day and between long day shifts (p-direction >0.98). Conversely, late chronotypes slept longer and better than early chronotypes between evening shifts (p-direction >0.98).

### Shift-cluster schedule composition and adverse sleep

The isotemporal substitution analysis revealed similar risk profiles for chronic insomnia (figure 2A) and short average sleep duration (figure 2B), although estimates were generally stronger for short sleep. Overall, reallocating day shifts to any other shift (rotating schedules) was associated with higher odds of chronic insomnia or short sleep, although most CI included 1.00. Interestingly, replacing day shifts with evening shifts was associated with increased odds of chronic insomnia (OR 1.44, 95% CI 1.12–1.85 for >30–50% and OR 1.55, 95% CI 1.12–2.13 for >50–70% evening shifts) and short sleep (OR 2.18, 95% CI 1.50–3.11 for >50–70% evening shifts). Participants working almost exclusively night shifts (>90%) were credibly more likely to report short sleep (OR 2.39, 95% CI 1.81–3.15) and chronic insomnia (OR 1.26, 95% CI 1.01–1.58). For the longer shift-clusters, workers reporting working >90% in long evening shifts were also more likely to report short sleep (OR 2.69, 95% CI 1.98–3.68), while those working >90% long day shifts were less likely to do so (OR 0.38, 95% CI 0.23–0.61).

Estimates from M1 and M4 (supplementary figures S5–S6) were similar to those presented in figure 2 (M2).

**Figure:**
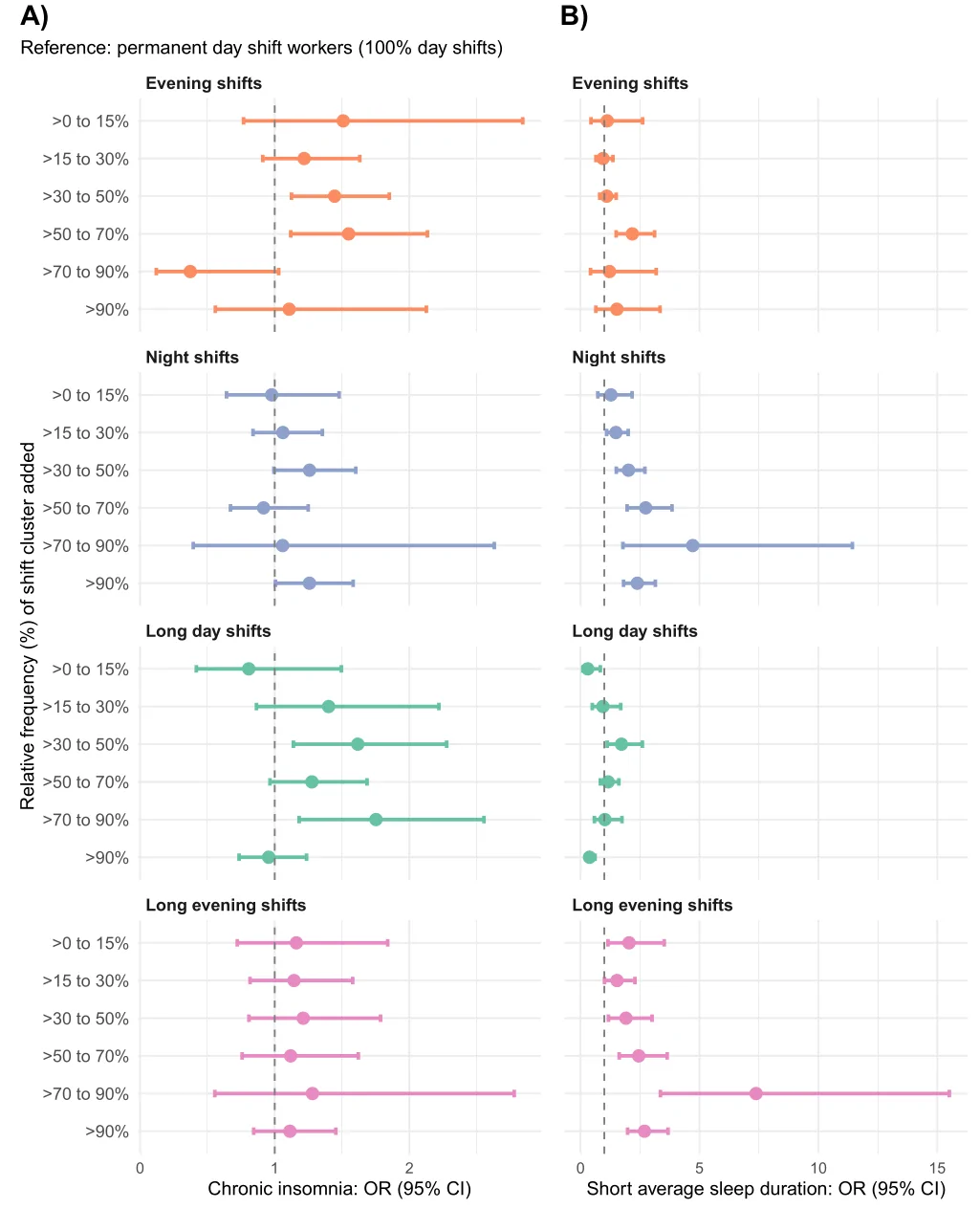
**Figure 2.** Estimated odds of A) chronic insomnia and B) short average sleep duration (<6 hours/day) when reallocating a given proportion (y-axis) of the day shifts to alternative shift types. Odds ratios (OR) and 95% Bayesian credible intervals (CI) represent the effect of an isotemporal substitution where shifts reallocated from day shifts to the shift type indicated on the y-axis, compared to permanent day shifts. All estimates are adjusted for age, gender chronotype, country of residence, education, income satisfaction, marital status, body mass index (scaled), alcohol consumption and smoking status (Model 2).

### Classic shift work metrics and adverse sleep

In the models on the association between classic night shift history and sleep outcomes (tables 2–3), working night shifts currently was associated with chronic insomnia (OR_M2_ 1.34, 95% CI 1.12–1.59) and short average sleep duration (OR_M2_ 2.72, 95% CI 2.12–3.52) after adjusting for confounders. Cumulative night shift work experience was also associated with adverse sleep outcomes, with higher odds associated with longer exposures.

**Table 2:** Association between classic shift work metrics and chronic insomnia. Results estimated using Bayesian logistic regression. **Statistically credible odds ratios (OR) are highlighted in bold.** [REF=reference; CI= credible interval; SD=standard deviation.]

	Chronic insomnia (N=6245)						
	Prevalence		Model 1 ^a^		Model 2 ^b^		Model 4 ^c^
	N (%)		OR (95% CI)		OR (95% CI)		OR (95% CI)
**Night shift history**							
Night shift work status							
Never	202 (17.6)		REF		REF		
Formerly	202 (20.9)		1.22 (0.99–1.51)		1.13 (0.91–1.41)		
Currently	935 (22.6)		**1.38 (1.17–1.63)**		**1.34 (1.12–1.59)**		
Years worked night shifts							
0	213 (17.9)		REF		REF		REF
≤5	423 (18.6)		1.08 (0.90–1.31)		1.06 (0.88–1.28)		0.97 (0.79–1.18)
6–15	393 (24.1)		**1.46 (1.22–1.77)**		**1.37 (1.12–1.66)**		1.19 (0.96–1.47)
≥16	310 (26.8)		**1.56 (1.28–1.92)**		**1.56 (1.26–1.94)**		**1.32 (1.04–1.68)**
**Current schedules**							
Schedule type							
Permanent day	326 (17.7)		REF		REF		REF
Permanent night	208 (20.4)		1.18 (0.97–1.43)		1.17 (0.96–1.43)		1.08 (0.87–1.34)
Rotating shifts	805 (23.8)		**1.45 (1.26–1.67)**		**1.42 (1.22–1.65)**		**1.28 (1.08–1.52)**
Frequency of night shifts (10% increase)			1.00 (0.99–1.02)		1.01 (0.99–1.02)		
Rotation speed							
Not applicable	534 (18.7)		REF		REF		
Daily change	176 (26.6)		**1.60 (1.31–1.94)**		**1.70 (1.38–2.11)**		
Change every 2–4 days	219 (23.6)		**1.35 (1.13–1.62)**		**1.32 (1.09–1.59)**		
Change every week	200 (22.9)		**1.26 (1.05–1.51)**		1.18 (0.98–1.43)		
Change every 2–3 weeks	48 (25.3)		**1.45 (1.03–2.03)**		1.34 (0.94–1.90)		
Rotation speed varies	126 (22.1)		**1.25 (1.00–1.55)**		**1.30 (1.03–1.63)**		
Other	36 (21.8)		1.18 (0.80–1.71)		1.11 (0.75–1.61)		
Weekly working hours (1 SD increase) ^d^			1.00 (0.94–1.07)		1.02 (0.95–1.10)		1.01 (0.94–1.08)

^a^ Adjusted for age and gender. ^b^ Model 1 + chronotype, country of residence, education, income satisfaction, marital status, body mass index (scaled), alcohol consumption and smoking status. ^c^ Model 2 + years worked on night shifts, schedule type, and weekly working hours added simultaneously to the model. ^d^ 1 SD = 6.20 hours.

**Table 3:** Association between classic shift work metrics and short average sleep duration (<6 hours/day). Results estimated using Bayesian logistic regression. **Statistically credible odds ratios (OR) are highlighted in bold.** [REF=reference; CI=credible interval; SD=standard deviation].

	Short average sleep duration (N=6245)						
	Prevalence		Model 1 ^a^		Model 2 ^b^		Model 4 ^c^
	N (%)		OR (95% CI)		OR (95% CI)		OR (95% CI)
**Night shift history**							
Night shift work status							
Never	75 (6.5)		REF		REF		
Formerly	89 (9.2)		**1.37 (1.00–1.89)**		1.32 (0.97–1.81)		
Currently	636 (15.4)		**2.55 (2.01–3.28)**		**2.72 (2.12–3.52)**		
Years worked on night shifts							
0	80 (6.7)		REF		REF		REF
≤5	275 (12.1)		**2.04 (1.58–2.66)**		**1.99 (1.53–2.59)**		**1.30 (1.00–1.70)**
6–15	245 (15.0)		**2.40 (1.85–3.10)**		**2.45 (1.89–3.20)**		**1.37 (1.05–1.82)**
≥16	200 (17.3)		**2.27 (1.73–2.99)**		**2.67 (2.02–3.58)**		**1.37 (1.02–1.86)**
**Current schedules**							
Schedule type							
Permanent day	114 (6.2)		REF		REF		REF
Permanent night	165 (16.2)		**2.80 (2.19–3.60)**		**2.87 (2.20–3.77)**		**2.33 (1.79–3.04)**
Rotating shifts	521 (15.4)		**2.74 (2.23–3.38)**		**3.15 (2.53–3.95)**		**2.60 (2.06–3.28)**
Frequency of night shifts (10% increase)			**1.08 (1.06–1.10)**		**1.09 (1.06–1.11)**		
Rotation speed							
Not applicable	279 (9.8)		REF		REF		
Daily change	99 (15)		**1.71 (1.33–2.19)**		**2.31 (1.76–3.02)**		
Change every 2–4 days	143 (15.4)		**1.69 (1.36–2.10)**		**1.96 (1.55–2.45)**		
Change every week	138 (15.8)		**1.62 (1.29–2.02)**		**1.67 (1.32–2.10)**		
Change every 2–3 weeks	49 (25.8)		**3.11 (2.19–4.37)**		**2.94 (2.00–4.25)**		
Rotation speed varies	71 (12.5)		**1.37 (1.04–1.80)**		**1.77 (1.33–2.35)**		
Other	21 (12.7)		1.30 (0.81–2.04)		1.35 (0.83–2.18)		
Weekly working hours (1 SD increase) ^d^			**1.14 (1.05–1.22)**		**1.13 (1.04–1.22)**		**1.09 (1.00–1.17)**

^a^ Adjusted for age and gender. ^b^ Model 1 + chronotype, country of residence, education, income satisfaction, marital status, body mass index (scaled), alcohol consumption and smoking status. ^c^ Model 2 + years worked on night shifts, schedule type, and weekly working hours added simultaneously to the model. ^d^ 1 SD = 6.20 hours.

Workers with rotating schedules had higher odds of chronic insomnia (OR_M2_ 1.42, 95% CI 1.22–1.65) and short sleep (OR_M2_ 3.15, 95% CI 2.53–3.95) compared to permanent day workers. Permanent night workers were more likely to report short sleep duration (OR_M2_ 2.87, 95% CI 2.20–3.77) compared to permanent day workers. Increases of about 6.20 hours (1 SD) in weekly working hours (OR_M2_ 1.13, 95% CI 1.04–1.22) or 10% in frequency of night shifts (OR_M2_ 1.09, 95% CI 1.06–1.11) were associated with higher odds of short sleep but not chronic insomnia. Among rotating workers, those reporting varying or quick rotation speeds had higher odds of chronic insomnia.

In models mutually adjusted for cumulative night shift experience, current shift schedule and weekly working hours (M4), OR were similar in direction as in M2, but estimates for years worked on night shifts and schedule type were markedly attenuated. Despite the structural relationship between these variables, multicollinearity was minimal (generalized variance inflation factors <1.3), suggesting the model could distinguish between variables.

Sensitivity analyses excluding participants with missing information in covariates (supplementary tables S3–S4) or using non-informative priors (supplementary tables S5–S6) yielded similar results. We did not observe large differences across chronotypes (supplementary tables S7–S8).

## Discussion

We analyzed a total of 12 104 shifts provided by 6245 participants in eight EU countries. Five data-driven shift clusters were identified, which were associated with differences in shift-specific sleep duration and sleep quality. Compared to permanent day workers, schedules consisting almost entirely of night shifts (>90%) and combinations of day and evening shifts were associated with higher odds of adverse sleep outcomes. We also analyzed classic shift work metrics and found that rotating schedules and longer cumulative exposure to shift work were associated with higher odds of chronic insomnia and short sleep, and longer weekly working hours with short sleep.

Three of the five shift clusters identified in this work overlapped considerably with the prespecified categories of day, evening and night shifts, but showed smaller within-group variability in shift timing and length than prespecified categories. The two remaining clusters – long day and long evening shifts– had a mean length of >10 hours. This highlights the importance of considering shift length, in addition to timing, as a key shift characteristic, and indicates that prespecified categories might fail to fully capture the diversity of work schedules and interrelations with overtime work. There are only a few studies using unsupervised learning methods, such as cluster (14, 15) or latent class analysis (16), to identify work schedule patterns and assess their association with health and safety outcomes. Unlike studies assessing single shift aspects [eg (7, 11–13),], unsupervised learning techniques can handle multiple features at once and identify real-world schedule patterns.

The data-driven clusters were associated with differences in shift-specific sleep duration and quality. Participants reported the longest and best quality sleep between evening shifts, and the shortest and poorest quality sleep between night shifts. These findings are consistent with a large body of literature (6, 12, 25–27). In this sample, long day shifts were also associated to longer and better sleep than (shorter) day shifts. Previous studies showed that, compared to 8h shifts, very long shifts (>16 hours) are associated with fatigue and poor sleep (10), while there seems to be little to no difference in sleep outcomes for 10–12-hour shifts (10, 28–30). Thus, in our sample, the longer and better sleep quality between long day shifts compared to between shorter day shifts might be explained by the later start of long day shifts, which could prolong sleep opportunity the night before. This is in line with previous findings among pilots (31), train-drivers (32) and industrial workers (32, 33). Likewise, sleep between long evening shifts was also credibly longer than between day shifts. Moreover, shift-specific sleep duration and quality varied by chronotype. Late chronotypes exhibited better sleep between evening shifts and between night shifts compared to earlier chronotypes, whereas early chronotypes reported better sleep between day shifts. Aligning work schedules with individual chronotype may improve sleep, particularly among extreme chronotypes (34). These findings highlight the need to account for inter-individual differences in circadian preference or phase and support the development of personalized prevention strategies.

While shift-specific sleep duration was longer between evening and long day shifts, substituting day shifts with either of them was associated with increased odds of chronic insomnia and short sleep. This could indicate that, while later start times might increase sleep opportunity on the day of the shift, rotating schedules might result in greater sleep irregularity and a reduction of the overall sleep duration and quality. Previous work has shown that sleep duration might be reduced immediately after a shift rotation (33); but our survey was not designed to capture sleep dynamics throughout a shift cycle. Furthermore, our models using classic shift work metrics, as well as a number of studies, show that rotating workers are indeed more likely to experience short sleep duration (27, 35) and chronic insomnia symptoms (7, 12, 36) than permanent day workers. Similarly and consistent with previous literature (12, 26, 27, 35, 37), in this analysis, (almost) permanent night shift work (>90%) was associated with higher odds of chronic insomnia and short average sleep duration. Workers reporting >90% of long evening shifts were also more likely to report short sleep, but those reporting >90% long day shifts had lower odds of short sleep. This, combined with the findings from the shift-specific sleep analysis, suggests that delaying the start time to the mid-morning may increase sleep opportunity, even if shift length is extended.

The analysis of classic shift work metrics was in line with the data-driven approach and previous literature, suggesting that prevalence of chronic insomnia and short sleep is higher among rotating shift workers (27) and current night shift workers (12, 26, 27, 36). Besides, odds of short sleep or chronic insomnia increased with longer cumulative exposure to night shift work. Two recent observational studies seem to suggest that risk of insomnia (38) and poor sleep quality (39) increase for up to 9–10 years of experience and then stabilize, albeit with considerable interindividual variation (38). In contrast, in our sample the prevalence of insomnia was highest in the group with the longest shift work exposure, with estimates remaining credible in mutually adjusted models, suggesting both cumulative shift work exposure and current shift work schedules may independently contribute to the risk of adverse sleep outcomes.

Differences in night shift assessments and definitions could explain some of the conflicting results in the associations of night shift work and sleep health, but also chronic diseases such as breast cancer (40). In our analysis, there was indeed some discrepancy between the prespecified night shift category and the night shift cluster. In addition, the reference groups varied in the two analytic approaches. While in the classic shift metrics models, the reference group (permanent day) comprised workers reporting only one shift schedule that did not include three hours of work between 00:00 and 05:00 (ie, it included also permanent evening workers), in the isotemporal models the reference group consisted of those working permanently in the day shift cluster. This was a more homogenous group of shifts lasting around 8 hours, starting in the morning and ending in the afternoon, which could have resulted in a “cleaner” reference group. Exposure misclassification is a common limitation of studies on shift work and its health effects (41). Comparisons that classify individuals simply as current or ever night workers versus non–night workers may misclassify exposure, as individuals working other non-standard schedules are often included in the reference group. Given that one third of the European workforce works on atypical hours (42), it is essential that future studies distinguish between different types of non-standard work schedules to more accurately estimate their associations with health outcomes.

Finally, we found that longer weekly working hours were associated with higher odds of short sleep, but not chronic insomnia. An increase of about 6 hours in weekly working hours was associated with 13% higher odds of short sleep. A systematic review of observational studies (8) showed that, in non-shift workers, long working hours (>40 hours/week or >8 hours/day) were associated with poorer sleep quality and shorter sleep duration. Moreover, interventions reducing total working hours while maintaining the salary have been shown to increase sleep duration and improve sleep habits (43).

### Strengths and limitations

This study has several strengths. First, it analyzed information from eight different European countries and different work sectors, which enabled a comprehensive description of distinct shift patterns. However, this diversity increased the variability in participants and schedules, so studies in specific populations should be conducted to confirm these findings. Second, the use of a data-driven clustering approach provided a more nuanced characterization of work schedules than traditional definitions, allowing simultaneous comparisons of shift length, start and end times. Third, to our knowledge, this is the first observational study that uses Bayesian statistical methods and isotemporal models to explore the complex relationships of different and related shift work metrics with adverse sleep outcomes. Fourth, the models were carefully adjusted for a comprehensive set of confounders and mediators, reducing the risk for residual confounding.

Nevertheless, several limitations must be acknowledged. First, our study relied on a large convenience sample and therefore may not be representative of the broader European (shift) working population. Consequently, other or less common shift patterns may not have been captured and our results may not reflect work schedules across all occupational settings or countries represented in our study population. Second, while the majority of our sample (57.9%) consists of shift workers from a diverse range of work sectors (eg, transport, logistics, or engineering), workers in the healthcare, social work, and beauty care sector represent the largest occupational group (42.1%). This broad sector representation enhances the generalizability of our findings compared to many existing shift-work studies that focus exclusively on a single industry or occupational group. Nonetheless, differences in industry-specific scheduling patterns may still exist, and future studies should explore work schedule patterns in cohorts with a more balanced distribution of work sectors. Third, the high survey attrition rate may have introduced selection bias if non-response was related to educational and socioeconomic level, workload and other factors associated with shift work and sleep. Fourth, workers experiencing greater difficulties related to shift work may have been more interested in completing the survey, potentially leading to an overestimation of the prevalence of chronic insomnia or short sleep and selection bias. However, because the survey was advertised as a general health survey among shift workers rather than specifically as a sleep survey, the potential for self-selection based primarily on sleep problems may have been reduced. Fifth, unlike studies drawing on payroll data (14, 15), shift schedules in this study were self-reported. While payroll records rarely include detailed information on shift-specific sleep and sleep outcomes, self-report methods introduce the risk of recall or desirability bias. This may have resulted in misclassification of shift exposures or underreporting of sleep problems and adverse health behaviors. In addition, because we relied on questionnaire-assessed schedules, we were unable to analyze the exact chronological sequence of shifts, nor did we have access to information on recovery times, or rest breaks during shifts. However, we sought to mitigate these constraints. While the broader literature frequently lacks both granular shift-work metrics and shift-specific sleep data, we summarized and characterized our respondents’ schedules using detailed shift-work metrics. This approach aligns with the consensus guidelines (44). Furthermore, whereas the majority of existing studies evaluate scheduling characteristics in isolation without looking into combinations of metrics, we addressed this gap by analyzing how these metrics co-occur, allowing for a much more comprehensive assessment of shift-work exposure within a survey framework. Sixth, there was no information on the number of days-off, therefore, average sleep duration was estimated under the assumption of a standard schedule of five working days and two days off per week. This assumption may have introduced measurement error in estimates of average sleep duration, particularly among workers with irregular schedules. Furthermore, the cross-sectional design precludes causal inference and does not rule out reverse causation, whereby individuals with pre-existing sleep problems may select or remain in certain shift schedules. Finally, as these analyses were exploratory, the results should be interpreted with caution and confirmed in further studies with objective measures of shift work schedules and sleep.

### Concluding remarks

We identified five data-driven shift clusters characterized by variations in shift timing and length. Shift-specific sleep duration and quality differed between clusters, with the shortest and poorest sleep occurring between night shifts. Participants working >90% night shifts, rotating schedules, or long weekly hours had greater odds of adverse sleep outcomes compared to permanent day workers. These findings emphasize that scheduling factors are key determinants of sleep health, thereby providing targets for interventions. The application of unsupervised learning in this large, diverse sample demonstrates that data-driven approaches can comprehensively capture the impact of real-world shift patterns on sleep and health.

## Supplementary material

Supplementary material 1 Supplementary material

## Data Availability

The complete questionnaire from the online survey is openly accessible (https://doi.org/10.5281/zenodo.17423134). The dataset and its codebook will be shared on reasonable request to the corresponding authors.
