# Supplementary material for: Shift work schedule patterns and sleep outcomes: results from the SHIFT2HEALTH survey across eight European countries
Source: Scand J Work Environ Health. 2026 Oct 26;52(6):702–13. doi: 10.5271/sjweh.4329 (PMC13738667; doi:10.5271/sjweh.4329)
Supplement: Supplementary file 1 [file SJWEH-52-6-702-s001.pdf]

Shift work schedule patterns and sleep outcomes: results from the SHIFT2HEALTH survey across eight European countries[1]

by Isabel Santonja, MBBS, MPH, [1] Coen Dros, MSc,[2] Sandra Haider, PhD,[3] Eva Winzer, PhD,[3] Maria Wakolbinger, Assoc. Prof., [3] Katrin Scionti, PhD,[4] Vanessa Schoissengeier, MSc,[4, 5] Magdalena A Wrzesińska, Prof.,[6] Katarzyna W Binder-Olibrowska, PhD,[6] Jarosław Rakoczy, MA,[6] Anna Lena Aufschnaiter, MSc,[5, 7] Christina Höfler, BSc,[7] Marlies Wallner, Prof.,[7] Catalina Cuparencu, Assistant Prof.,[8] Karl-Heinz Wagner, Prof.,[4] Hendriek C Boshuizen, PhD,[9] Monique H Vingerhoeds, PhD,[10] Heidi Lammers-van der Holst, Assistant Prof.,[2] Kyriaki Papantoniou, Assoc. Prof. [1,11]

1. Supplementary materials
2. Correspondence to: Kyriaki Papantoniou, Medical University of Vienna, Centre for Public Health, Department of Epidemiology, Vienna, Austria. [E-mail: kyriaki.papantoniou@meduniwien.ac.at]
   Isabel Santonja, Medical University of Vienna, Centre for Public Health, Department of Epidemiology, Vienna, Austria. [E-mail: Isabel.santonja@meduniwien.ac.at]

Contents

Section 1. Methods 3

Figure S1. Flow chart of participants included in the study. 3

Figure S2. Diagnostic metrics for Van Mises Fisher clustering solutions of 1 to 10 components (k) 4

Figure S3. Directed acyclic graph of the association between shift schedules and sleep. 5

Appendix 1. Bayesian Model Specification and Prior Selection 6

Table S1. Summary of Prior Distributions. 7

Section 2. Results 8

Figure S4. Sleep duration (A) and quality (B) across chronotypes within shift clusters 8

Figure S5. Estimated odds of A) chronic insomnia and B) short average sleep duration (<6 h/day) when reallocating a given proportion of day shifts to alternative shift types..........9

Figure S6. Estimated odds of A) chronic insomnia and B) short average sleep duration (<6 h/day) reallocating a given proportion of day shifts to alternative shift types. 10

Table S2. Sociodemographic and health-related characteristics of the study sample. 11

Table S3. Association between shift work metrics and chronic insomnia excluding participants with missings in covariates 13

Table S4. Association between shift work metrics and short average sleep duration (<6h/day) excluding participants with missings in covariates 14

Table S5 Association between shift work metrics and chronic insomnia using non-informative priors 15

Table S6. Association between shift work metrics and short average sleep duration (<6h/day) using non-informative priors 16

Table S7. Association between shift work metrics and chronic insomnia across chronotype categories 17

Table S8. Association between shift work metrics and short average sleep duration (< 6h/day) across chronotype categories 18

References 48

Section 1. Methods

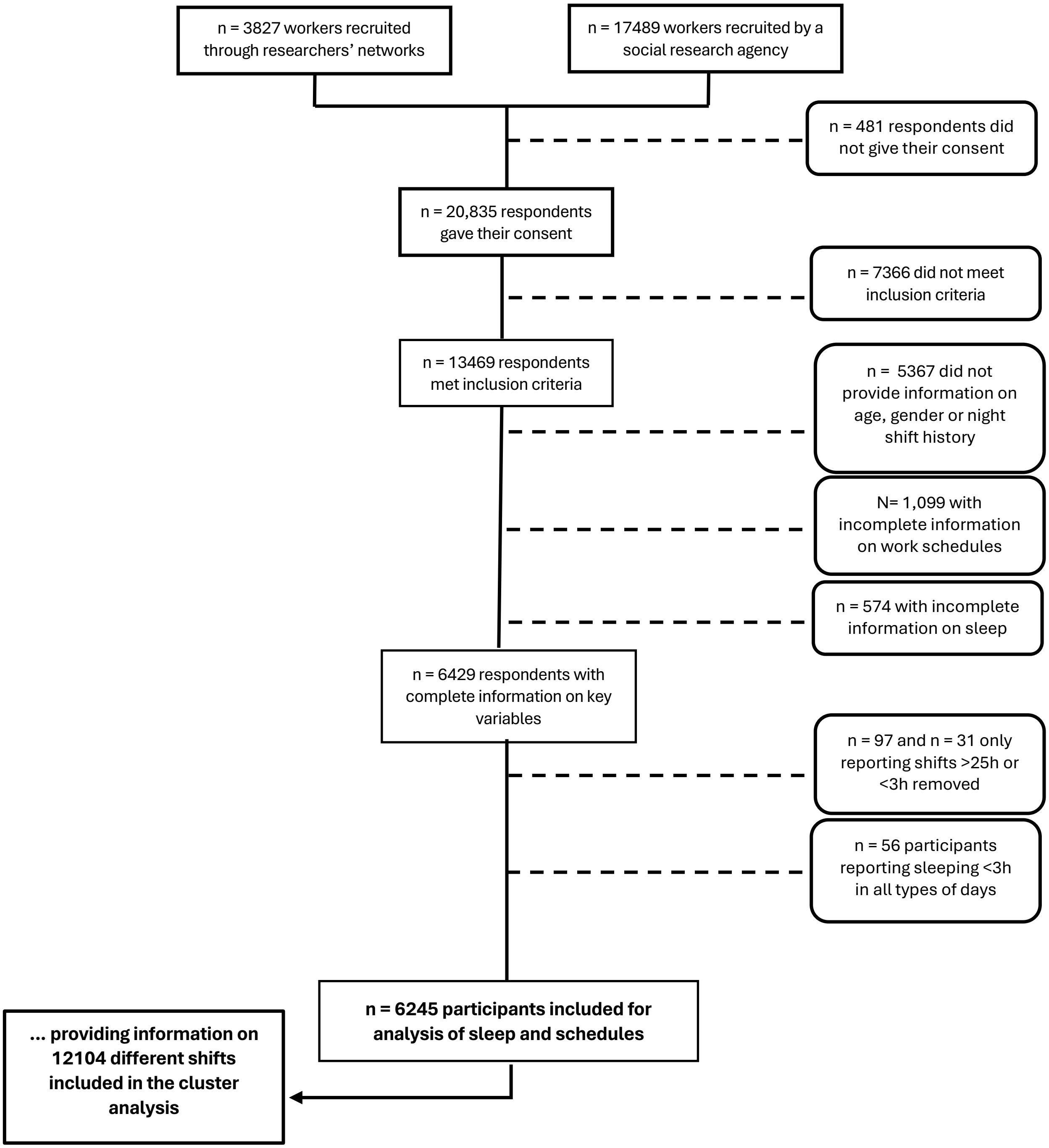

Figure S1. Flow chart of participants included in the study.

Figure S2. Diagnostic metrics for Van Mises Fisher clustering solutions of 1 to 10 components (k). A) Bayesian Information Criterion (BIC), B) Sillhoutte score, C) Log-likelihood and D) Average Adjusted Rand Index (ARI) for k = 1 to k = 10. The dashed lines indicates the optimal value (i.e. global minima for BIC, and global maxima for silhouette scores, log-likelihood and average ARI) for each of the metrics.

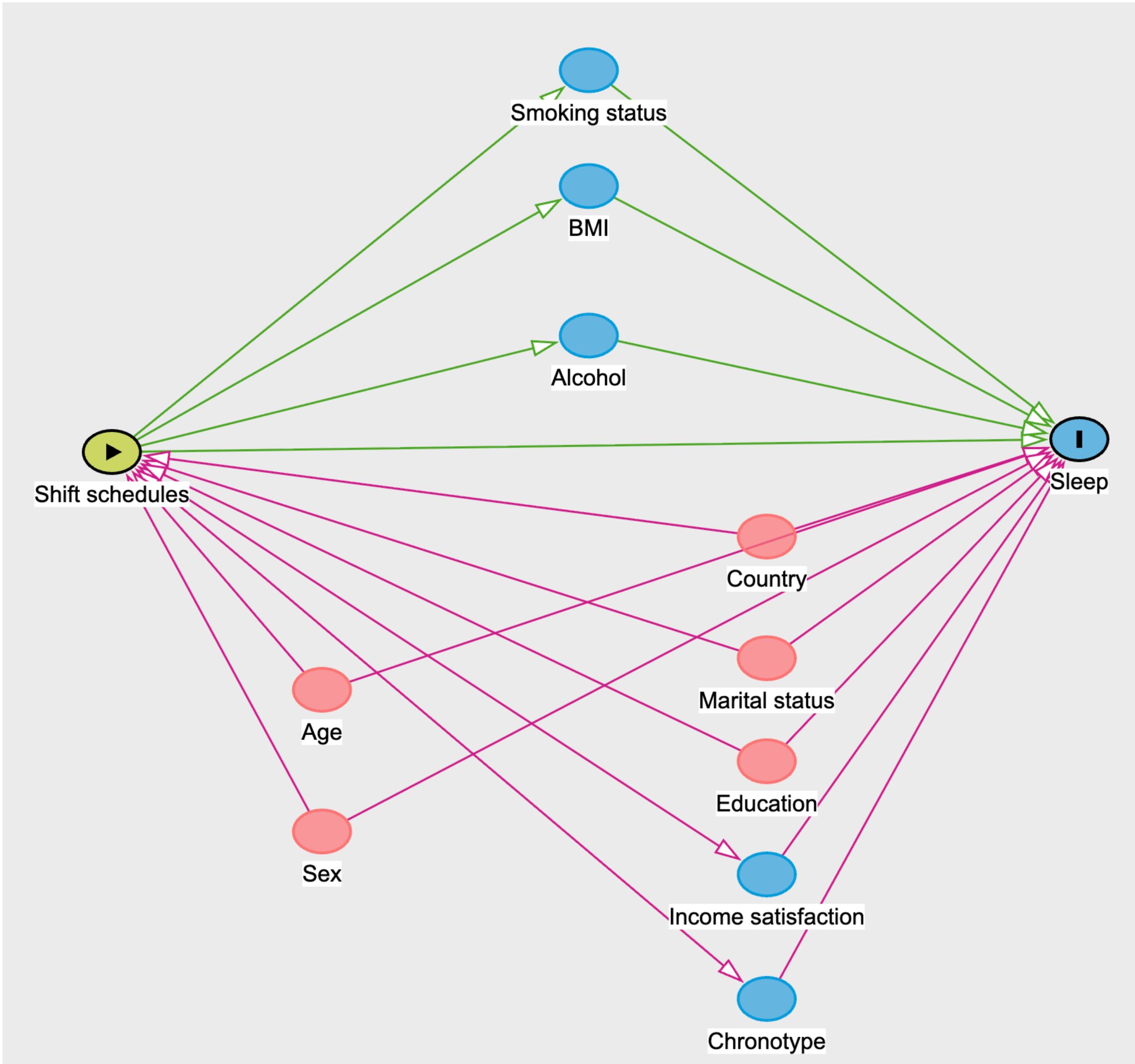

Figure S3. Directed acyclic graph of the association between shift schedules and sleep. "Shift schedules" (light green node with a triangle icon) represents the exposure variables and "Sleep" (blue node with an 'I' icon) represents the exposures, linked by hypothesized direct and indirect directional pathways (green arrows). Blue nodes ("Smoking status", "BMI", "Alcohol") represent potential intermediate variables (or mediators). Pink nodes with pink arrows ("Age", "Sex", "Country", "Marital status", "Education") denote confounding variables. Blue nodes with pink arrows ("Income satisfaction", "Chronotype") represented variables that could be considered both intermediate or confounding variables.

Appendix 1. Bayesian Model Specification and Prior Selection

A Bayesian framework was employed 1) to calculate sleep duration and quality distributions on days of different shift clusters accounting for multiple measurements and 2) to estimate the associations between night shift history, current schedule characteristics and schedule composition with sleep outcomes.

All models were fitted using the *brms* R-package (1) with the *cmdstanr* backend and with four Markov chains, each run for 4000 iterations (2000 warm-up, 2000 sampling), and a random seed of 647 to ensure reproducibility.

Weakly informative priors (*Table S1*) were selected for the models with chronic insomnia or short average sleep duration as outcomes, in order to provide slight regularization, preventing the models from over-fitting while remaining broad enough to allow the data to dominate the posterior distribution. For intercepts in our logistic models, "prior-informed" distributions were used, based on established prevalence rates of insomnia and short sleep duration in the literatura. (2-4)

For the linear regression models, a Student-t likelihood was chosen to mitigate the influence of potential outliers in sleep duration. This adds a "heavy-tail" component to the model, ensuring that extreme individual values do not disproportionately bias the population-level estimates.

Prior predictive checks confirmed plausible outcome ranges, and model convergence (R-hat < 1.01) and fit (posterior predictive checks, leave-one-out cross-validation) were verified.

Table S1. Summary of Prior Distributions.

Model Type	Parameter	Prior Distribution	Source
Linear mixed-effects (Shift-associated Sleep Duration)	Intercept	Student-t(3, 7, 2.5)	
	Covariates	Normal(0, 1)	
	Residual SD (σ)	Student-t(3, 0, 5)	
	Degrees of Freedom (ν)	Gamma(2, 0.1)	
	Subject Variance (sd)	Student-t(3, 0, 2.5)	
Ordinal mixed-effects model	Intercept	Student-t(3, 0, 2.5)	
	Covariates	Flat	
	Subject Variance (sd)	Student-t(3, 0, 2.5)	
Logistic Regression	Intercept (Insomnia)	Normal(-2.2, 1)	Prevalence estimates (2, 3)
	Intercept (Short Sleep)	Normal(-2.7, 1)	Prevalence estimates (1)
	Main exposures (classic metrics or relative frequencies	Normal(0, 1)	
	Other covariates	Normal(0, 0.5)	
Sensitivity logistic regression models (non-informative priors)	Intercept	Normal(0, 10)	
	Main Exposures	Normal(0, 10)	
	Other covariates	Normal(0, 100)	

SD = Standard deviation

Section 2. Results

Figure S4. Sleep duration (A) and quality (B) across chronotypes within shift clusters (N=12104 shifts from 6245 subjects). All estimates were derived from Bayesian multilevel models accounting for repeated measures within subjects. Day shifts: reference category (Ref.). * and ** indicate a 99% probability of reporting sleep being longer/of better quality or shorter/of worse quality, respectively, compared to early chronotypes within shift clusters. A) The violin plots represent the observed distribution of sleep duration for each chronotype across shift clusters. Overlaid on each violin is the Bayesian posterior predictive interval: the central dot represents the posterior median, the thick bar indicates the 50% credible interval (CI), and the thin lines represent the 95% CI. B) Diverging stacked bars representing the probability of sleep quality categories.

____**Figure S5. Estimated odds of A) chronic insomnia and B) short average sleep duration (<6 h/day) when reallocating a given proportion of day shifts to alternative shift types.** Odds Ratios (OR) and 95% Bayesian Credible Intervals (CI) represent the effect of an isotemporal substitution where shifts reallocated from day shifts to the shift type indicated on the y-axis, compared to permanent day shifts. All estimates are adjusted for age and gender (Model 1).

Figure S6. Estimated odds of A) chronic insomnia and B) short average sleep duration (<6 h/day) reallocating a given proportion of day shifts to alternative shift types. Odds Ratios (OR) and 95% Bayesian Credible Intervals (CI) represent the effect of an isotemporal substitution where shifts reallocated from day shifts to the shift type indicated on the y-axis, compared to permanent day shifts. All estimates are adjusted for age, gender chronotype, country of residence, education, income satisfaction, marital status, BMI (scaled), alcohol consumption, smoking status, years worked on night shifts and total weekly working hours (Model 3).

Table S2. Sociodemographic and health-related characteristics of the study sample.

	N = 6245	
	**N (%)**	**Mean (SD)**
**Age** (years)	–	40.6 (10.7)
**Gender**		
Female	3165 (50.7%)	–
Male	3060 (49.0%)	–
Other	20 (0.3%)	–
**Country**		
Austria	996 (15.9%)	–
The Netherlands	840 (13.5%)	–
Poland	805 (12.9%)	–
Germany	785 (12.6%)	–
Spain	718 (11.5%)	–
Greece	716 (11.5%)	–
Denmark	632 (10.1%)	–
Italy	605 (9.7%)	–
Other	53 (0.8%)	–
Unknown	95 (1.5%)	–
**Work sector**		
Health, social work, beauty care	2628 (42.1%)	–
Trade, logistics, transport	507 (8.1%)	–
Office, marketing, finance, law, security	481 (7.7%)	–
Electrical engineering, electronics, telecommunications, IT	320 (5.1%)	–
Other	2309 (37.0%)	–
**Education**		
Basic education	388 (6.2%)	–
High School and Vocational Training	1532 (24.5%)	–
Advanced Training and College	1528 (24.5%)	–
University Degrees and beyond	2758 (44.2%)	–
Other	39 (0.6%)	–
**Income satisfaction**		
Finding it very difficult on present income	306 (4.9%)	–
Finding it difficult on present income	1042 (16.7%)	–
Coping on present income	2959 (47.4%)	–
Living comfortably on present income	1914 (30.6%)	–
Prefer not to say	24 (0.4%)	–
**Marital status**		
Single	1683 (26.9%)	–
Married/living -together with my partner	4161 (66.6%)	–
Divorced, separated, or partner has died	401 (6.4%)	–
**Chronotype**		
Definitely morning	1062 (17.0%)	–
Rather morning	1335 (21.4%)	–
Intermediate	1511 (24.2%)	–
Rather evening	1561 (25.0%)	–

	N = 6245	
	**N (%)**	**Mean (SD)**
**BMI**	–	26.0 (5.3)
**Alcohol consumption (glasses/week)**		
None	1821 (29.2%)	–
1 drink	1180 (18.9%)	–
2-3 drinks	1454 (23.3%)	–
4-5 drinks	683 (10.9%)	–
6-9 drinks	525 (8.4%)	–
10 or more drinks	387 (6.2%)	–
Unknown	195 (3.1%)	–
**Smoking history**		
Current smoker	2265 (36.3%)	–
Former smoker	1352 (21.6%)	–
Never smoker	2433 (39.0%)	–
Unknown	195 (3.1%)	–

Table S3. Association between shift work metrics and chronic insomnia excluding participants with missing information on covariates (n =5942).

	Prevalence [N (%)]	Model 1[a] OR (95% CI)	Model 2[b] OR (95% CI)	Model 4[c] OR (95% CI)
Night shift history				
**Night shift work status**				
Never	202 (17.6%)	REF	REF	-
Formerly	202 (20.9%)	1.23 (0.99-1.53)	1.14 (0.92-1.44)	-
Currently	935 (22.6%)	**1.38 (1.17-1.64)**	**1.35 (1.14-1.62)**	**-**
**Years worked on night shifts**				
0 years	213 (17.9%)	REF	REF	REF
up to 5 years	423 (18.6%)	1.09 (0.91-1.31)	1.07 (0.89-1.29)	0.98 (0.79-1.20)
6-15 years	393 (24.1%)	**1.48 (1.23-1.79)**	**1.38 (1.14-1.68)**	1.21 (0.98-1.52)
16+ years	310 (26.8%)	**1.57 (1.28-1.93)**	**1.58 (1.27-1.97)**	**1.35 (1.06-1.75)**
Current schedules				
**Schedule type**				
Permanent day	326 (17.7%)	REF	REF	REF
Permanent night	208 (20.4%)	1.18 (0.97-1.43)	1.18 (0.97-1.45)	1.08 (0.87-1.34)
Rotating shifts	805 (23.8%)	**1.45 (1.26-1.68)**	**1.43 (1.22-1.66)**	**1.28 (1.08-1.52)**
**Frequency of night shifts (10% increase)**	–	1.00 (0.99-1.02)	1.00 (0.99-1.02)	**-**
**Rotation speed**				
Not applicable	534 (18.7%)	REF	REF	**-**
Daily change	176 (26.6%)	**1.62 (1.33-1.96)**	**1.72 (1.39-2.11)**	**-**
Change every 2-4 days	219 (23.6%)	**1.36 (1.14-1.63)**	**1.32 (1.10-1.61)**	**-**
Change every week	200 (22.9%)	**1.27 (1.06-1.53)**	1.18 (0.97-1.44)	**-**
Change every 2-3 weeks	48 (25.3%)	**1.47 (1.03-2.05)**	1.35 (0.93-1.92)	**-**
Rotation speed varies	126 (22.1%)	**1.26 (1.01-1.56)**	**1.31 (1.04-1.67)**	**-**
Other	36 (21.8%)	1.19 (0.82-1.72)	1.11 (0.73-1.64)	**-**
**Weekly working hours (1 SD increase)[d]**	–	1.00 (0.94-1.07)	1.02 (0.96-1.09)	1.01 (0.94-1.08)

Results are based on Bayesian logistic regression analyses excluding participants with missing values in covariates (n =5942). Statistically credible Odds Ratios are highlighted in bold.
OR: Odds Ratio. CI: credible interval. SD: standard deviation.
a. Model 1, adjusted for age and gender.
b. Model 2: additionally adjusted for chronotype, country of residence, education, income satisfaction, marital status, BMI (scaled), alcohol consumption and smoking status.
c. Model 4: model 2, with the variables years worked on night shifts, schedule type and weekly working hours being added simultaneously to the model.
d. 1 SD = 6.13 hours.

Table S4. Association between shift work metrics and short average sleep duration (<6h/day) excluding participants with missing information on covariates (n = 5942).

	Prevalence [N (%)]	Model 1[a] OR (95% CI)	Model 2[b] OR (95% CI)	Model 4[c] OR (95% CI)
Night shift history				
**Night shift work status**				
Never	73 (6.7%)	REF	REF	-
Formerly	85 (9.4%)	**1.39 (1.01-1.91)**	1.31 (0.94-1.83)	-
Currently	606 (15.4%)	**2.49 (1.96-3.23)**	**2.65 (2.05-3.46)**	-
**Years worked on night shifts**				
0 years	78 (6.9%)	REF	REF	REF
up to 5 years	262 (12.1%)	**1.99 (1.54-2.57)**	**1.95 (1.50-2.54)**	1.28 (0.98-1.67)
6-15 years	234 (15.2%)	**2.38 (1.84-3.08)**	**2.41 (1.84-3.15)**	**1.36 (1.02-1.79)**
16+ years	190 (17.2%)	**2.19 (1.65-2.91)**	**2.55 (1.90-3.43)**	1.33 (0.98-1.80)
Current schedules				
**Schedule type**				
Permanent day	111 (6.3%)	REF	REF	
Permanent night	155 (15.9%)	**2.69 (2.09-3.45)**	**2.74 (2.10-3.63)**	**2.24 (1.70-2.95)**
Rotating shifts	498 (15.5%)	**2.68 (2.19-3.33)**	**3.07 (2.45-3.85)**	**2.54 (2.03-3.23)**
**Frequency of night shifts (10% increase)**	-	**1.08 (1.06-1.10)**	**1.09 (1.06-1.11)**	-
**Rotation speed**				
Not applicable	266 (9.8%)	REF	REF	-
Daily change	94 (15.2%)	**1.74 (1.34-2.24)**	**2.34 (1.77-3.07)**	-
Change every 2-4 days	132 (14.9%)	**1.63 (1.31-2.02)**	**1.87 (1.46-2.37)**	-
Change every week	134 (16.1%)	**1.65 (1.32-2.06)**	**1.70 (1.35-2.13)**	-
Change every 2-3 weeks	47 (25.7%)	**3.08 (2.16-4.38)**	**2.92 (1.99-4.24)**	-
Rotation speed varies	70 (12.9%)	**1.41 (1.05-1.85)**	**1.81 (1.35-2.42)**	-
Other	21 (13.5%)	1.35 (0.82-2.13)	1.42 (0.85-2.30)	-
**Weekly working hours (1 SD increase)[d]**	-	**1.14 (1.06-1.23)**	**1.14 (1.05-1.23)**	**1.10 (1.01-1.19)**

Results are based on Bayesian logistic regression analyses excluding participants with missing values in covariates (n =5942). Statistically credible Odds Ratios are highlighted in bold.
OR: Odds Ratio. CI: credible interval. SD: standard deviation.
a. Model 1, adjusted for age and gender.
b. Model 2: additionally adjusted for chronotype, country of residence, education, income satisfaction, marital status, BMI (scaled), alcohol consumption and smoking status.
c. Model 4: model 2, with the variables years worked on night shifts, schedule type and weekly working hours being added simultaneously to the model.
e. 1 SD = 6.13 hours.

Table S5 Association between shift work metrics and chronic insomnia using non-informative priors (n = 6245).

	Prevalence [N (%)]	Model 1[a] OR (95% CI)	Model 2[b] OR (95% CI)	Model 4[c] OR (95% CI)
Night shift history				
**Night shift work status**				
Never	202 (17.6%)	REF	REF	-
Formerly	202 (20.9%)	1.23 (0.99-1.53)	1.14 (0.92-1.44)	-
Currently	935 (22.6%)	**1.38 (1.17-1.64)**	**1.35 (1.14-1.62)**	**-**
**Years worked on night shifts**				
0 years	213 (17.9%)	REF	REF	REF
up to 5 years	423 (18.6%)	1.09 (0.91-1.31)	1.07 (0.89-1.29)	0.98 (0.79-1.20)
6-15 years	393 (24.1%)	**1.48 (1.23-1.79)**	**1.38 (1.14-1.68)**	1.21 (0.98-1.52)
16+ years	310 (26.8%)	**1.57 (1.28-1.93)**	**1.58 (1.27-1.97)**	**1.35 (1.06-1.75)**
Current schedules				
**Schedule type**				
Permanent day	326 (17.7%)	REF	REF	REF
Permanent night	208 (20.4%)	1.18 (0.97-1.43)	1.18 (0.97-1.45)	1.08 (0.87-1.34)
Rotating shifts	805 (23.8%)	**1.45 (1.26-1.68)**	**1.43 (1.22-1.66)**	**1.28 (1.08-1.52)**
**Frequency of night shifts (10% increase)**	–	1.00 (0.99-1.02)	1.00 (0.99-1.02)	**-**
**Rotation speed**				
Not applicable	534 (18.7%)	REF	REF	**-**
Daily change	176 (26.6%)	**1.62 (1.33-1.96)**	**1.72 (1.39-2.11)**	**-**
Change every 2-4 days	219 (23.6%)	**1.36 (1.14-1.63)**	**1.32 (1.10-1.61)**	**-**
Change every week	200 (22.9%)	**1.27 (1.06-1.53)**	1.18 (0.97-1.44)	-
Change every 2-3 weeks	48 (25.3%)	**1.47 (1.03-2.05)**	1.35 (0.93-1.92)	-
Rotation speed varies	126 (22.1%)	**1.26 (1.01-1.56)**	**1.31 (1.04-1.67)**	**-**
Other	36 (21.8%)	1.19 (0.82-1.72)	1.11 (0.73-1.64)	**-**
**Weekly working hours (1 SD increase)[d]**	–	1.00 (0.94-1.07)	1.02 (0.96-1.09)	1.01 (0.94-1.08)

Results are based on Bayesian logistic regression analyses using non-informative priors. Statistically credible Odds Ratios are highlighted in bold.

OR: Odds Ratio. CI: credible interval. SD: standard deviation.

a. Model 1, adjusted for age and gender.

b. Model 2: additionally adjusted for chronotype, country of residence, education, income satisfaction, marital status, BMI (scaled), alcohol consumption and smoking status.

c. Model 4: model 2, with the variables years worked on night shifts, schedule type and weekly working hours being added simultaneously to the model.

d. 1 SD = 6.20 hours.

Table S6. Association between shift work metrics and short average sleep duration (<6h/day) using non-informative priors (n = 6245).

	Prevalence [N (%)]	Model 1[a] OR (95% CI)	Model 2[b] OR (95% CI)	Model 4[c] OR (95% CI)
Night shift history				
**Night shift work status**				
Never	75 (6.5%)	REF	REF	-
Formerly	89 (9.2%)	**1.41 (1.02 - 1.94)**	1.35 (0.97-1.89)	-
Currently	636 (15.4%)	**2.59 (2.03 - 3.35)**	**2.82 (2.18-3.68)**	**-**
**Years worked on night shifts**				
0 years	80 (6.7%)	REF	REF	REF
up to 5 years	275 (12.1%)	**2.11 (1.62-2.76)**	**2.08 (1.59-2.73)**	1.32 (0.99-1.76)
6-15 years	245 (15.0%)	**2.49 (1.90-3.25)**	**2.58 (1.98-3.41)**	**1.39 (1.02-1.88)**
16+ years	200 (17.3%)	**2.35 (1.77-3.12)**	**2.84 (2.10-3.86)**	**1.40 (1.01-1.94)**
Current schedules				
**Schedule type**				
Permanent day	114 (6.2%)	REF	REF	REF
Permanent night	165 (16.2%)	**2.88 (2.24-3.70)**	**2.97 (2.27-3.90)**	**2.58 (1.93-3.45)**
Rotating shifts	521 (15.4%)	**2.80 (2.26-3.45)**	**3.29 (2.64-4.13)**	**2.87 (2.24-3.68)**
**Frequency of night shifts (10% increase)**	**-**	**1.08 (1.06-1.10)**	**1.09 (1.07-1.11)**	**-**
**Rotation speed**				
Not applicable	279 (9.8%)	REF	REF	**-**
Daily change	99 (15%)	**1.75 (1.35-2.25)**	**2.46 (1.87-3.20)**	**-**
Change every 2-4 days	143 (15.4%)	**1.73 (1.39-2.13)**	**2.04 (1.61-2.59)**	**-**
Change every week	138 (15.8%)	**1.65 (1.32-2.05)**	**1.72 (1.37-2.17)**	-
Change every 2-3 weeks	49 (25.8%)	**3.25 (2.28-4.60)**	**3.11 (2.14-4.51)**	-
Rotation speed varies	71 (12.5%)	**1.40 (1.05-1.84)**	**1.87 (1.38-2.54)**	**-**
Other	21 (12.7%)	1.33 (0.81-2.11)	1.42 (0.85-2.28)	**-**
**Weekly working hours (1 SD increase)[d]**	**-**	**1.14 (1.05-1.22)**	**1.13 (1.04-1.22)**	**1.08 (1.00-1.17)**

Results are based on Bayesian logistic regression analyses using non-informative priors. Statistically credible Odds Ratios are highlighted in bold.

OR: Odds Ratio. CI: credible interval. SD: standard deviation.

a. Model 1, adjusted for age and gender.

b. Model 2: additionally adjusted for chronotype, country of residence, education, income satisfaction, marital status, BMI (scaled), alcohol consumption and smoking status.

c. Model 4: model 2, with the variables years worked on night shifts, schedule type and weekly working hours being added simultaneously to the model.

d. 1 SD = 6.20 hours.

Table S7. Association between shift work metrics and chronic insomnia across chronotype categories (n = 6 245).

	**Early chronotype (n = 2397)**		**Intermediate chronotype (n = 1511)**		**Late chronotype (n = 2337)**	
	**Prevalence [N (%)]**	**M2-OR (95% CI)[a]**	**Prevalence [N (%)]**	**M2-OR (95% CI)[a]**	**Prevalence [N (%)]**	**M2-OR (95% CI)[a]**
Night shift history						
**Night shift work status**						
Never	85 (16.4%)	REF	39 (13.5%)	REF	78 (22.9%)	REF
Formerly	69 (17.6%)	0.99 (0.70-1.41)	51 (21.2%)	1.49 (0.94-2.37)	82 (24.5%)	1.05 (0.72-1.52)
Currently	306 (20.6%)	**1.43 (1.09-1.88)**	223 (22.7%)	**1.74 (1.22-2.55)**	406 (24.4%)	1.11 (0.83-1.49)
**Years worked on night shifts**						
0 years	89 (16.7%)	REF	42 (14.0%)	REF	82 (23.1%)	REF
up to 5 years	152 (17.3%)	1.12 (0.83-1.51)	91 (17.5%)	1.28 (0.86-1.89)	180 (20.7%)	0.87 (0.65-1.18)
6-15 years	111 (19.3%)	1.26 (0.92-1.73)	86 (24.6%)	**1.82 (1.20-2.74)**	196 (27.8%)	1.22 (0.90-1.66)
16+ years	108 (26.4%)	**1.66 (1.19-2.33)**	94 (27.6%)	**2.07 (1.32-3.19)**	108 (26.4%)	1.28 (0.90-1.83)
Current schedules						
**Schedule type**						
Permanent day	142 (17.6%)	REF	67 (14.4%)	REF	117 (20.7%)	REF
Permanent night	57 (15.1%)	0.99 (0.70-1.38)	49 (20.3%)	1.50 (0.98-2.24)	102 (25.4%)	1.22 (0.88-1.68)
Rotating shifts	261 (21.5%)	**1.35 (1.07-1.71)**	197 (24.5%)	**1.89 (1.38-2.58)**	347 (25.3%)	**1.32 (1.03-1.69)**
**Frequency of night shifts (10% increase)**	-	1.00 (0.97-1.03)	-	1.02 (0.98-1.05)	-	1.01 (0.98-1.04)
**Rotation speed**						
Not applicable	199 (16.8%)	REF	116 (16.4%)	REF	219 (22.6%)	REF
Daily change	61 (25.6%)	**1.93 (1.35-2.71)**	37 (24.0%)	**1.73 (1.10-2.71)**	78 (28.9%)	**1.49 (1.07-2.05)**
Change every 2-4 days	60 (18.1%)	1.07 (0.76-1.48)	57 (27.4%)	**1.78 (1.20-2.59)**	102 (26.2%)	1.28 (0.96-1.69)
Change every week	69 (21.9%)	1.23 (0.89-1.69)	52 (24.8%)	1.45 (0.97-2.11)	79 (22.8%)	1.02 (0.74-1.39)
Change every 2-3 weeks	13 (20.0%)	1.13 (0.60-2.08)	14 (28.0%)	1.70 (0.87-3.24)	21 (28.0%)	1.19 (0.69-2.01)
Rotation speed varies	41 (21.1%)	1.47 (0.99-2.16)	31 (20.8%)	1.34 (0.84-2.14)	54 (23.9%)	1.16 (0.80-1.64)
Other	17 (24.3%)	1.39 (0.76-2.47)	6 (18.2%)	1.06 (0.43-2.43)	13 (21.0%)	0.87 (0.45-1.61)
**Weekly working hours (1 SD increase)[b]**	–	0.98 (0.87-1.10)	–	1.07 (0.92-1.23)	–	1.04 (0.93-1.15)

Results are based on Bayesian logistic regression analyses. Statistically credible Odds Ratios are highlighted in bold.
OR: Odds Ratio. CI: credible interval. SD: standard deviation.
a. M2: Model 2, adjusted for age, gender, country of residence, education, income satisfaction, marital status, BMI (scaled), alcohol consumption and smoking status. b. 1 SD = 6.20 hours.

Table S8. Association between shift work metrics and short average sleep duration (< 6h/day) across chronotype categories (n = 6 245).

	Early chronotype (n = 2397)		Intermediate chronotype (n = 1511)		Late chronotype (n = 2337)	
	Prevalence [N (%)]	M2-OR (95% CI)[a]	Prevalence [N (%)]	M2-OR (95% CI)[a]	Prevalence [N (%)]	M2-OR (95% CI)[a]
Night shift history						
**Night shift work status**						
Never	29 (5.6%)	REF	22 (7.6%)	REF	24 (7.0%)	REF
Formerly	36 (9.2%)	1.50 (0.91-2.47)	22 (9.1%)	1.05 (0.58-1.91)	31 (9.3%)	1.21 (0.71-2.09)
Currently	196 (13.2%)	**2.68 (1.81-4.03)**	172 (17.5%)	**2.46 (1.58-3.90)**	268 (16.1%)	**2.72 (1.80-4.20)**
**Years worked on night shifts**						
0 years	30 (5.6%)	REF	24 (8.0%)	REF	26 (7.3%)	REF
up to 5 years	98 (11.1%)	**2.11 (1.40-3.19)**	66 (12.7%)	**1.50 (0.95-2.46)**	111 (12.8%)	**1.94 (1.26-3.00)**
6-15 years	74 (12.9%)	**2.49 (1.62-3.82)**	56 (16.0%)	**1.88 (1.17-3.10)**	115 (16.3%)	**2.38 (1.55-3.70)**
16+ years	59 (14.4%)	**2.36 (1.50-3.78)**	70 (20.6%)	**2.92 (1.77-4.90)**	71 (17.4%)	**2.25 (1.40-3.66)**
Current schedules						
**Schedule type**						
Permanent day	43 (5.3%)	REF	35 (7.5%)	REF	36 (6.4%)	REF
Permanent night	50 (13.2%)	**3.02 (1.98-4.62)**	36 (14.9%)	**1.96 (1.20-3.20)**	79 (19.7%)	**3.14 (2.08-4.81)**
Rotating shifts	168 (13.8%)	**3.19 (2.29-4.50)**	145 (18.0%)	**2.83 (1.93-4.24)**	208 (15.2%)	**3.16 (2.19-4.61)**
**Rotation speed**						
Not applicable	93 (7.9%)	REF	71 (10.0%)	REF	115 (11.9%)	
Daily change	26 (10.9%)	**1.74 (1.08-2.76)**	20 (13.0%)	**1.76 (1.00-2.99)**	53 (19.6%)	**2.73 (1.83-4.00)**
Change every 2-4 days	41 (12.4%)	**1.74 (1.16-2.59)**	48 (23.1%)	**2.61 (1.70-3.98)**	54 (13.9%)	**1.55 (1.08-2.22)**
Change every week	53 (16.8%)	**2.11 (1.42-3.07)**	41 (19.5%)	**1.97 (1.26-3.01)**	44 (12.7%)	1.17 (0.79-1.70)
Change every 2-3 weeks	16 (24.6%)	**2.97 (1.59-5.55)**	12 (24.0%)	**2.07 (1.01-4.10)**	21 (28.0%)	**2.88 (1.61-5.05)**
Rotation speed varies	20 (10.3%)	1.58 (0.93-2.64)	21 (14.1%)	1.69 (0.96-2.88)	30 (13.3%)	**1.67 (1.06-2.59)**
Other	12 (17.1%)	**2.13 (1.09-3.96)**	3 (9.1%)	0.83 (0.28-2.17)	6 (9.7%)	0.96 (0.40-2.09)
**Weekly working hours (1 SD increase)[b]**	-	1.10 (0.96-1.26)	-	**1.37 (1.17-1.61)**	-	1.02 (0.89-1.16)

Results are based on Bayesian logistic regression analyses. Statistically credible Odds Ratios are highlighted in bold.

OR: Odds Ratio. CI: credible interval. SD: standard deviation.

a. M2: Model 2, adjusted for age, gender, country of residence, education, income satisfaction, marital status, BMI (scaled), alcohol consumption and smoking status.

b. 1 SD = 6.20 hours.

References

1. Bürkner P-C. brms: An R Package for Bayesian Multilevel Models Using Stan. Journal of Statistical Software. 2017;80(1):1 - 28.
2. Kocevska D, Lysen TS, Dotinga A, Koopman-Verhoeff ME, Luijk M, Antypa N, et al. Sleep characteristics across the lifespan in 1.1 million people from the Netherlands, United Kingdom and United States: a systematic review and meta-analysis. Nat Hum Behav. 2021;5(1):113-22.
3. Chalet FX, Albanese E, Egea Santaolalla C, Ellis JG, Ferini-Strambi L, Heidbreder A, et al. Epidemiology and burden of chronic insomnia disorder in Europe: an analysis of the 2020 National Health and Wellness Survey. J Med Econ. 2024;27(1):1308-19.
4. Morin CM, Jarrin DC. Epidemiology of Insomnia: Prevalence, Course, Risk Factors, and Public Health Burden. Sleep Med Clin. 2022;17(2):173-91.
